# Imaging Familial and Sporadic Neurodegenerative Disorders Associated with Parkinsonism

**DOI:** 10.1007/s13311-020-00994-4

**Published:** 2021-01-11

**Authors:** David J. Brooks

**Affiliations:** 1 Department of Nuclear Medicine, Aarhus University, Aarhus N, 8200 Denmark; 2 Translational and Clinical Research Institute, Newcastle University, Newcastle upon Tyne, NE4 5PL UK

**Keywords:** Parkinson, Atypical, Genetic, MRI, PET, SPECT

## Abstract

In this paper, the structural and functional imaging changes associated with sporadic and genetic Parkinson’s disease and atypical Parkinsonian variants are reviewed. The role of imaging for supporting diagnosis and detecting subclinical disease is discussed, and the potential use and drawbacks of using imaging biomarkers for monitoring disease progression is debated. Imaging changes associated with nonmotor complications of PD are presented. The similarities and differences in imaging findings in Lewy body dementia, Parkinson’s disease dementia, and Alzheimer’s disease are discussed.

**Supplementary Information:**

The online version contains supplementary material available at 10.1007/s13311-020-00994-4.

## Imaging Modalities

### Magnetic Resonance Imaging

Water protons behave like magnetic dipoles that become aligned in a strong static magnetic field like tiny bar magnets and precess around their axis at the Larmor frequency which is in the radiofrequency range. Pulses of electromagnetic radiation at the Larmor frequency can flip these proton dipoles through 180° to an unstable excited state which then relaxes back to the baseline state emitting the absorbed radiation. Magnetic resonance imaging (MRI) detects these water proton relaxation signals both longitudinal (T1) and transverse (T2) to the static magnetic field which are then reconstructed to produce images of brain grey and white matter. MRI can quantitate brain tissue volume and detect structural abnormalities.

Susceptibility-weighted imaging (SWI) detects raised brain levels of paramagnetic iron from the increased relaxation rates present. Iron levels are high in the substantia nigra reticulata (SNr), the globus pallidus, and the red nuclei. Nigrosomes are small clusters of dopaminergic cells within the substantia nigra compacta (SNc) exhibiting calbindin negativity on immunohistochemical staining. Nigrosome-1 is located in the posterior third of the SNc and returns a high signal on SWI. The healthy nigrosome-1 appearance is like a “swallow tail” on 3 T-SWI and this is lost in Parkinson’s disease (PD) [1]. SWI can also detect the neuromelanin present in normal adult substantia nigra compacta (SNc) and locus ceruleus (LC) as it binds paramagnetic iron [2]. In atypical PD cases, SWI detects the abnormal iron deposition present in the basal ganglia [3].

Diffusion tensor imaging (DTI) is sensitized to measure the amplitude and direction of water proton diffusion along nerve fibers enabling alterations in the structural connectivity of the brain to be visualized. DTI is achieved by pulsing a magnetic field gradient to disperse the orientation of water proton dipoles and then pulsing a second field gradient in the opposite direction to refocus the dipoles. This refocusing is only perfect if no movement of water has occurred between the opposing field gradient pulses and loss of expected signal allows the direction (fractional anisotropy) and amplitude (diffusivity) of water flow to be computed. DTI allows nerve fiber tracts to be visualized (tractography) and the damage to the structural connectivity of basal ganglia and cortex to be detected in parkinsonian conditions.

Functional MRI detects changes in the oxygen level of blood draining in brain venules. The iron in deoxyhemoglobin is paramagnetic until it binds oxygen so blood oxygen–dependent (BOLD) sequences can monitor the slow regional oscillatory changes in venular oxygenation in the resting brain. Detection of brain regions which show synchronized oscillations of their venous oxygenation at rest provides evidence of functional connectivity. Independent component analysis (ICA) can identify intrinsic connectivity networks (ICNs) which facilitate attention, executive actions, movement, vision, and hearing [4]. In addition, a default mode network (DMN) connecting mesial premotor and temporoparietal areas can be seen at rest which is inactive on action. Loss of connectivity of these functional networks can be detected in the presence of neurodegenerative disease.

### Molecular Imaging

Positron emission tomography (PET) and single-photon emission computed tomography (SPECT) are both radiotracer-based imaging modalities. PET has higher sensitivity than SPECT, being able to detect femtomolar levels of positron-emitting radioisotopes at a spatial resolution of 2 to 4 mm. It allows quantitative *in vivo* examination of alterations in regional cerebral blood flow (rCBF), glucose, oxygen, and dopa metabolism and the availability of monoaminergic and other receptors. It can also detect abnormally aggregated β-amyloid and tau proteins. Most PET studies involve ^11^C- or ^18^F-labelled tracers. These 2 positron-emitting isotopes have short half-lives (20 and 110 min). ^11^C-labelled ligands need to be prepared on-site, requiring a cyclotron and hot cells. ^18^F-labelled ligands are also made on-site though some can be obtained from local distributing centers.

SPECT is cheaper and more widely available than PET. SPECT tracers are usually labeled with either ^123^I or ^99m^Tc, and these gamma-emitting isotopes have longer half-lives (13 and 6 h) than ^11^C or ^18^F so tracers can be purchased commercially. The SPECT tracers HMPAO and ECD are available for measuring regional brain blood flow whereas ^123^I-fluopane (DaTSCAN, GE Healthcare, Chicago, USA) and ^123^I-IBZM measure dopamine transporter and D2 receptor availability.

Magnetic resonance spectroscopy (MRS) is nonradiation based but has a lower sensitivity and spatial resolution than the radioisotope imaging approaches. Proton MRS can detect millimolar levels of *N*-acetylaspartate, lactate, phospholipids, and ATP at a spatial resolution of around 1 cm.

In summary, molecular imaging approaches can be used to detect *in vivo* the brain metabolic and perfusion changes associated with different parkinsonian disorders and dysfunction of dopaminergic, noradrenergic, serotonergic, and cholinergic neurotransmission.

## Fluid Biomarkers

Neuroimaging is complex and expensive so the availability of fluid biomarkers that could 1) distinguish Parkinson’s disease from healthy controls and essential tremors, 2) discriminate the different parkinsonian syndromes, and 3) predict rates of disease progression and the likelihood of superadded dementia would be welcome. Categories of biomarkers that have been investigated in studies on PD cerebrospinal fluid (CSF) include dopamine and its metabolites, other neurotransmitters, markers of oxidative stress, inflammation and immunological activity, growth factors, and abnormally aggregated proteins involved in typical and atypical PD pathologies [5].

To date, these studies have had some success. CSF dopamine levels are normally low and difficult to detect reliably though the development of single molecule array (SIMOA) technology could improve this situation. Aggregated α-synuclein (Asyn) protein is a component of the Lewy pathology that characterizes Parkinson’s disease. Low levels of raised Asyn aggregates are present in PD CSF and can now be detected using real-time quaking induced conversion (RT-QuIC) which provides an Asyn seed that attracts Asyn oligomers present in CSF to become attached [6]. After 1 to 2 days the aggregated Asyn can be detected with a fluorescent thioflavin T dye. This approach has a high sensitivity and specificity for detecting PD and dementia with Lewy body (DLB) cases. Raised aggregated Asyn can also be detected in blood exosomes of PD patients though the normal and PD ranges overlap [7].

Plasma levels of the protein neurofilament light (NfL) provide a nonspecific marker of axonal damage in neurodegenerations. Using SIMOA, raised levels are seen in PD which increase longitudinally correlating with cognitive deterioration [8]. Higher plasma NfL levels can be detected in the atypical parkinsonian syndromes multiple system atrophy (MSA) and progressive supranuclear palsy (PSP) compared to PD [9].

Raised CSF levels of P-tau and reduced levels of β-amyloid 1-42 are markers of the presence of Alzheimer pathology in the brains of Parkinson’s disease cases and predictors of their subsequent cognitive decline. Raised plasma P-tau 181 detectable with SIMOA is also a marker of the presence of brain Alzheimer pathology [10]. Raised CSF inflammatory and oxidative stress markers are seen in PD cases, but these are relatively nonspecific as they are also seen in Alzheimer cases.

In summary, while the field of fluid biomarkers is advancing rapidly, to date, none of these biomarkers are being routinely assayed to support a diagnosis of PD though this may well change in the near future.

## Parkinson’s Disease

The pathology of late-onset idiopathic Parkinson’s disease (PD) and dominantly inherited genetic variants is characterized by intraneuronal inclusions called Lewy bodies (LBs) and Lewy neurites which contain abnormally aggregated α-synuclein (Asyn) protein [11, 12]. LBs target the dopamine cells in the substantia nigra compacta (SNc) and the midbrain ventral tegmentum (VTA). SNc degeneration is associated with loss of striatal dopamine terminals, the dorsal putamen being worst affected, resulting in limb bradykinesia, rigidity, and rest tremor [13, 14]. VTA cell dysfunction leads to a loss of frontal and anterior cingulate dopamine terminals and impaired performance of executive tasks. Cholinergic cells are also targeted, projections from the pedunculopontine nucleus (PPN) to the thalamus mediating gait and balance whereas those in the nucleus basalis of Meynert (NBM) send projections to the cortex and facilitate memory, attention, and perception [15]. Lewy pathology also involves serotonergic cells in the median raphe and noradrenergic cells in the locus ceruleus [13] resulting in sleep disorders, such as daytime somnolence [16] and REM sleep behavior disorder (RBD) [17], and depression [18]. Lewy bodies can also be detected in limbic and association cortical areas, and if patients live for 20 years, around 80% of PD cases will develop later dementia (PDD) [19].

Whereas the majority of PD patients have an idiopathic disorder, a minority of PD cases are genetic in origin and a number of autosomal dominant and recessive susceptibility genes have now been identified. Mutations of the glucocerebrosidase (GBA) gene are the most common genetic determinant of PD in Caucasians, and in some populations, up to 30% of “sporadic” cases are heterozygous carriers of a GBA mutation [20]. Homozygous and compound heterozygous GBA mutation carriers develop Gaucher’s disease, a lysosomal storage disorder. The Gcase enzyme clears aggregated Asyn, but this mechanism fails when mutations are present. The second most common cause of dominantly inherited PD is a G2019S mutation of the lysine-rich repeat kinase 2 (LRRK2) gene. LRRK2 disease mimics late-onset sporadic PD and is present in 40% of North African Arab and 30% of Ashkenazi Jewish PD cases whereas only 5% of Caucasian cases carry the G2019S mutation [21]. The first familial cause of PD discovered was mutations and duplications of the α-synuclein (SNCA) gene [22]. Monomeric Asyn is concentrated in nerve terminals and regulates synaptic plasticity. Abnormally aggregated Asyn can be transmitted in a prion-like fashion forming Lewy bodies. SNCA mutations are associated with early-onset autosomal-dominant PD and Lewy body dementia. However, SNCA mutations are very rare compared to LRRK2 and GBA mutations.

Autosomal recessive Parkinson’s disease is severe and early onset but rarely associated with Lewy body pathology. The most common cause is homozygous or compound heterozygous mutations of the Parkin gene which is a ubiquitin ligase again involved in the clearance of aggregated proteins [23]. Patients typically present in their teens or 20s with lower limb dystonia and parkinsonism, but it can be later onset. Levodopa-induced dyskinesias occur early and are severe while nonmotor problems are less of an issue than in late-onset sporadic disease. Other rarer causes of recessive PD include homozygous or compound heterozygous mutations of the PINK1 and DJ1 genes.

## Imaging the Presynaptic Dopaminergic System

**Fig. 1:**
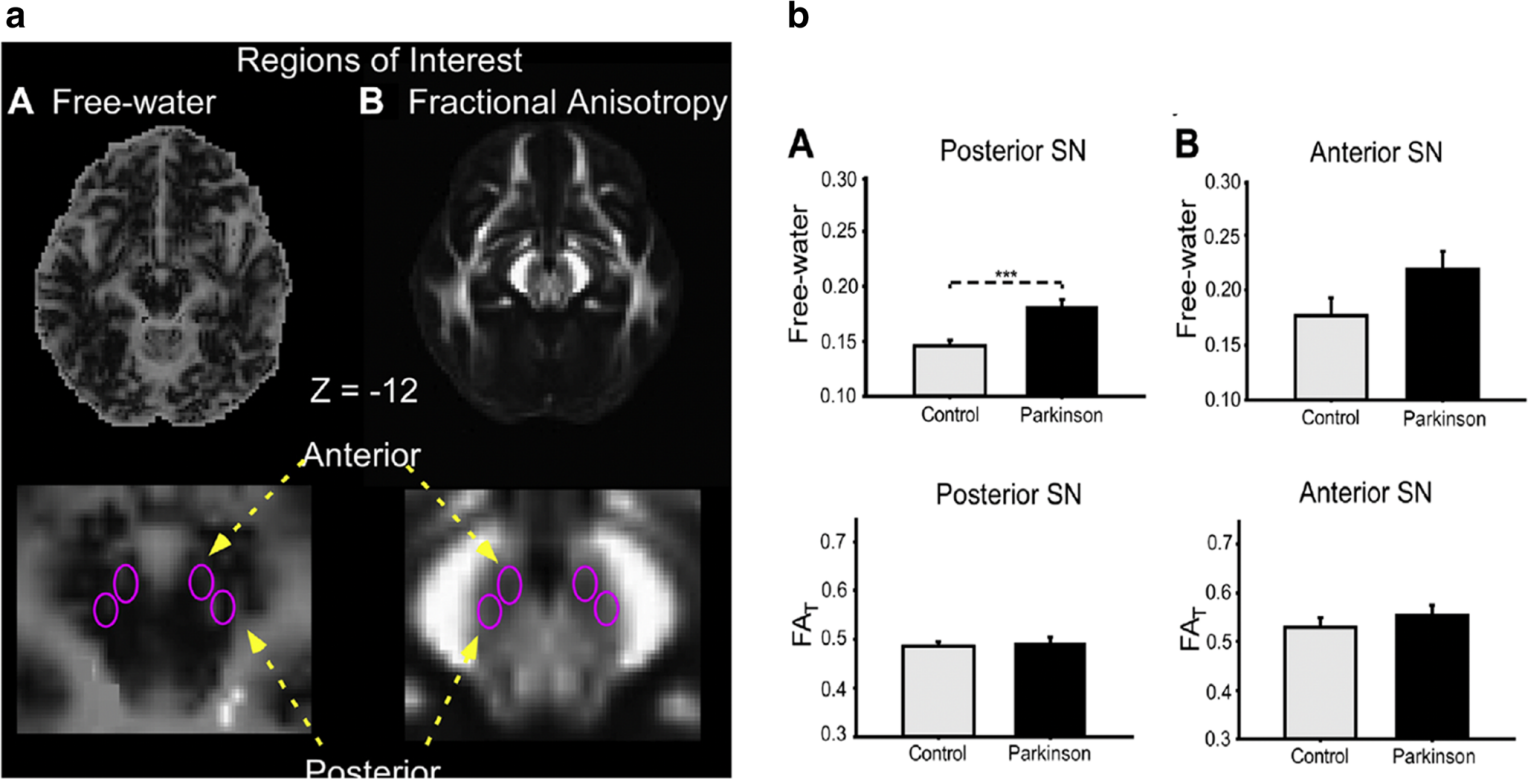
(a/b) Bitensor DTI of the midbrain. The substantia nigra free water pool is increased in Parkinson’s disease. (Picture from ref [26]: Ofori et al. 2015)

Ideally, one would like to be able to image the abnormal Asyn aggregates present in PD neurons. Small-molecule ligands such as BF227 exist which bind to these, but when tagged with an ^11^C or ^18^F isotope to make positron emission tomography (PET) tracers, they give low specific signals and they also bind as avidly to extracellular amyloid deposits [24]. As a consequence, imaging in PD aims to detect the consequences of the Asyn pathology. The integrity of the cell bodies in the substantia nigra compacta (SNc) can be imaged structurally with MRI while striatal DA terminal function can be imaged with radiotracer-based PET and SPECT. High field MRI detects a loss of nigrosome 1 signal in the ventrolateral SNc of PD cases [1] while MR sequences sensitive to paramagnetic neuromelanin also show ventrolateral signal loss [2, 25]. Using a bitensor approach, diffusion tensor imaging (DTI) is able to measure the size of the SNc free water pool and has shown that this is significantly increased in PD due to the cell loss [26] (Fig. 1). A 4-year follow-up of the Parkinson’s Progression Markers Initiative (PPMI) PD cohort showed that the posterior nigral free water pool increased along with locomotor disability on the Hoehn and Yahr scale [27].

**Fig. 2:**
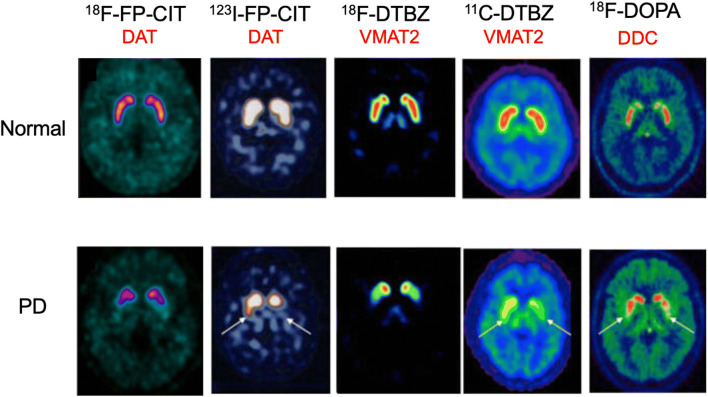
SPECT and PET images of dopamine terminal function in normal subjects and early PD patients

The function of striatal dopamine terminals in PD can be examined *in vivo* by examining dopamine transporter (DAT) and monoamine vesicle transporter (VMAT2) availability with either PET or SPECT or dopamine storage capacity with ^18^F-dopa PET [28]. DAT availability can be assessed with ^123^I-FP-CIT SPECT (DaTSCAN) [29] or ^18^F-FP-CIT PET [30]. VMAT2 density in dopamine terminals can be determined with ^11^C- or ^18^F-dihydrotetrabenazine (DTBZ) PET [31] (Fig. 2). Early PD cases show bilaterally reduced dorsal putamen dopamine terminal function, the activity being lowest in the putamen contralateral to the more affected limbs, while head of caudate and ventral striatal function are relatively spared. This characteristic pattern of signal loss is seen in both idiopathic and genetic causes of late-onset PD cases [32, 33]. Levels of putamen dopaminergic function correlate inversely with locomotor rigidity and bradykinesia. Patients with young-onset recessive PD generally show a more severe, global, and symmetrical loss of striatal dopamine terminal function expected given their degree of disability [34, 35].

PET and SPECT are able to detect subclinical dopaminergic loss in the putamen ipsilateral to the unaffected limb of early hemi-parkinsonian cases. It has been estimated that in late-onset disease, clinical parkinsonism occurs when PD patients have lost 40 to 50% of their dopamine terminal function in the posterior dorsal putamen, the most targeted striatal subregion [36]. In series in which clinically probable PD patients have been compared with essential tremor (ET) cases, imaging their dopamine function with PET and SPECT differentiated these conditions with a sensitivity and specificity greater than 90% [37, 38]. Given this, an abnormal PET or SPECT scan can be valuable for supporting a diagnosis of PD in which there is diagnostic doubt. Several studies have now examined the influence DAT imaging can have on the management of cases with uncertain parkinsonism (CUPS) [39]. While the pathology of these cases was not available, the CUPS study found that baseline SPECT findings agreed well with the clinical diagnosis assigned after 2 years of clinical follow-up. DAT imaging findings led to a change of baseline clinical diagnosis and management in 50% of these grey cases.

The Parkinson’s Progression Markers Initiative (PPMI) study has examined whether the finding of normal dopaminergic function with DAT SPECT excludes a diagnosis of PD in those thought to have clinical features—so-called subjects without evidence of dopamine deficiency (SWEDDs). In the PPMI series, 5 of 53 SWEDDs converted to clinical PD over 2 years of follow-up. However, retrospective review of their baseline scans suggested 4 may have been initially misclassified (J Seibyl; May 2019 PPMI Investigators Meeting). In the PRECEPT trial, 91 SWEDDs were followed up over 4 years and showed no significant clinical deterioration or loss of striatal ^123^I-beta-CIT uptake [40]. In a Scottish series, 150 cases of possible PD who had normal FP-CIT SPECT at baseline were also followed for 2 years. Only 3% of these subjects were still felt to have possible PD at their 2-year assessment [41]. Many of these subjects were reclassified as having a benign essential or dystonic tremor while other diagnoses included small-vessel disease and drug-induced syndromes. In general, a finding of normal presynaptic dopaminergic function on imaging is associated with a good prognosis in cases of suspected PD whatever their ultimate diagnosis.

While putamen dopamine terminal function measured with PET and SPECT shows an inverse correlation with severity of contralateral limb bradykinesia and rigidity in PD, rest tremor severity correlates poorly with dopaminergic status [29, 42]. This finding suggests that loss of nigrostriatal dopaminergic function may not be directly responsible for tremor onset in PD and helps to explain the variable response of tremor to dopaminergic replacement.

While all imaging biomarkers of dopamine terminal function are reduced in the putamen of symptomatic PD cases, DAT markers such as ^11^C-methylphenidate PET are most reduced while ^18^F-dopa uptake is least reduced. Putamen uptake of ^11^C-DTBZ, a marker of VMAT2 binding, is reduced to a level between ^18^F-dopa and ^11^C-methylphenidate [43]. This finding suggests that surviving putamen DA terminals in PD compensate by relatively increasing their levels of dopa decarboxylase to promote dopamine turnover and reducing their DAT availability to maintain synaptic transmitter levels.

## Neuroimaging Premotor PD

This topic has been recently reviewed by Barber and colleagues [44]. Postmortem studies have suggested that for every patient who develops clinical Parkinson’s disease, there are at least 10 subclinical cases with incidental Lewy body disease. Whereas the greatest risk factor for PD remains aging, other factors increasing the risk of developing Parkinson’s disease include having a family history, carriage of known susceptibility genes such as certain GBA mutations, late-onset idiopathic hyposmia, idiopathic REM sleep behavior disorder (RBD), and severe late-onset constipation.

In a Dutch series, 40 elderly relatives of PD patients who had no overt parkinsonism but who were hyposmic on olfactory screening were investigated with baseline beta-CIT SPECT and then followed clinically. Seven of these 40 relatives showed reduced ^123^I-beta-CIT uptake in one or more striatal subregions at baseline, and the 4 with lowest DAT binding subsequently converted to clinical PD over a 2-year follow-up period [45]. These findings suggest that, while DAT SPECT is capable of detecting preclinical dopaminergic dysfunction when present in at-risk subjects for PD, it has a low short-term positive predictive value in idiopathic hyposmia cases.

Asymptomatic carriers of LRRK2 and GBA gene mutations have been investigated with PET. DAT imaging proved the most sensitive marker of subclinical disease in LRRK2 disease, ^11^C-methylphenidate PET detecting significant reductions in the putamen signal in 2 of 3 asymptomatic carriers of the gene while their ^18^F-dopa PET was normal [32]. In a larger series of symptomatic and asymptomatic LRRK2 carriers, Wile and colleagues found that the symptomatic cases showed similar striatal loss of DAT binding and ^18^F-dopa uptake to idiopathic PD cases who had an equivalent disease duration [46]. The asymptomatic LRRK2 carriers also showed reduced DAT binding with ^11^C-methylphenidate PET, but their ^18^F-dopa uptake was normal. Striatal VMAT2 binding, measured with ^11^C-DTBZ PET, was reduced in all parkinsonian LRRK2 carriers but in only one asymptomatic carrier.

^18^F-dopa PET has, however, detected subclinical disease in other series of asymptomatic adult LRRK2 [47], GBA [33], and heterozygous parkin mutation carriers [48], relatives of cases with known familial PD [49], and identical cotwins of PD patients [50]. Three of 8 asymptomatic relatives in PD kindreds who were found to have reduced putamen ^18^F-dopa uptake developed clinical parkinsonism over a 5-year follow-up period.

Genetic causes of PD are also associated with changes in other neurotransmitter systems that are known to become deranged in the sporadic disease. Using ^11^C-PMP PET, a marker of acetylcholine esterase activity, raised cholinergic function was noted in the cortex of 16 asymptomatic LRRK2 carriers compared with healthy controls while idiopathic PD cases showed reduced cortical signal [51]. The symptomatic LRRK2 carriers showed normal and raised cortical and thalamic ^11^C-PMP uptake significantly higher than seen in idiopathic PD. These workers suggested that rises in cholinergic activity in LRRK2 carriers might represent a compensatory response to LRRK2-related dysfunction. Hypothalamic serotonin transporter (SERT) binding has also been reported to be raised in nonmanifesting LRRK2 carriers compared to healthy controls [46]. Brainstem ^11^C-DASB binding was elevated in nonmanifesting LRRK2 carriers relative to the parkinsonian carriers who had similar ^11^C-DASB binding to idiopathic PD. In contrast, A53T SNCA mutation carriers—both premotor and parkinsonian—showed reduced hypothalamic, striatal, and brainstem SERT binding even though the level of striatal DAT binding was still normal in the asymptomatic cohort [52]. This finding suggests that asymptomatic LRRK2 carriers do not provide a representative model of prodromal idiopathic PD, say, for use in drug trials of protective agents.

REM sleep behavior disorder is a prodromal phenotype of both idiopathic PD and of dementia with Lewy bodies (DLB). 45% of these cases convert to either PD or DLB over an 11-year follow-up [53]. Across different series, subclinical loss of putamen ^18^F-dopa uptake or DAT binding can be detected in a majority of RBD cases suggesting they could provide a suitable population for trialing the protective efficacy of putative neuroprotective agents for PD. In a prospective study of 20 RBD cases, 10 subjects had reduced striatal DAT binding at baseline and 13 after 3 years [54]. Three of the RBD cases converted to PD over 3 years.

FDG (^18^F-2-fluoro-2-deoxyglucose) PET is a marker of brain hexokinase activity which reflects levels of regional cerebral glucose metabolism (rCMRGlc). The uptake of FDG is governed primarily by neuronal synaptic activity, but also microglial activity if pathology is present. In nondemented PD patients, absolute levels of striatal and cortical rCMRGlc generally lie within the normal range but covariance analysis reveals an abnormal profile of relatively increased lentiform nucleus and reduced frontoparietal metabolism [55]. This has been labeled the “PD-related profile (PDRP),” and its degree of expression correlates with levels of motor disability rated with the Unified Parkinson’s Disease Rating Scale (UPDRS) [56]. Successful treatment with either dopaminergic replacement therapy or deep brain stimulation normalizes the PDRP [57–59]. RBD cases have also been investigated with FDG PET and the blood flow marker ECD SPECT to detect abnormal network activity [60]. Both of these imaging modalities detected PDRP expression by the RBD cases, and 8 of them subsequently converted to PD over a 5-year follow-up. Those converters showed the highest expression of the PDRP at baseline. Meles and colleagues studied 21 RBD cases confirmed on polysomnography with FDG PET [61]. They found a distinct idiopathic RBD (iRBD) metabolic profile which overlapped with the PDRP pattern described by Eidelberg and coworkers. This iRBD pattern was also expressed by PD patients irrespective of whether they also had symptoms of RBD. However, the iRBD pattern was most noticeable in PD-mild cognitive impairment (PD-MCI) cases suggesting its expression in PD is related to more extensive disease.

Recently, studies with ^11^C-PK11195 PET, a marker of microglial activation, and ^11^C-donepezil PET, a marker of acetylcholinesterase activity, have reported that RBD cases show subclinical inflammation in their substantia nigra, substantia inomminata, and occipital cortex [62] and a reduction of cholinergic function in their frontal and temporal cortex. This helps to explain why they convert to a more aggressive PD phenotype.

## Following PD Progression

Imaging DA terminal function has been used as a biomarker to follow PD progression and to monitor the efficacy of putative neuroprotective and restorative agents. A limitation is that these biomarkers only give information about one aspect of the disease and that their use assumes that the therapeutic interventions being tested have no direct effect on uptake of the imaging agent itself. This assumption has proved to be problematic in practice. Chronic exposure of the patient to drugs thought to have neuroprotective potential, such as MAOB inhibitors and dopamine agonists, but which also influence dopaminergic transmission, can potentially confound the use of imaging agents that are markers of dopamine storage or transporters.

^18^F-dopa PET and beta-CIT DAT SPECT series initially reported a 9% and 11% annual decline from baseline in the putamen dopamine terminal function of early PD patients over 2 to 4 years [63, 64]. These PD cases were all exposed to dopamine agonists and levodopa during the course of both these series. Subsequently, a slower 5% rate of decline was reported for early PD cases treated with a placebo over 1 year in a trial of the JNK inhibitor CEP1347 [65]. Following these initial observations, a series of trials of putative neuroprotective drugs followed using either ^18^F-dopa PET or DAT SPECT as biomarkers of their efficacy. The REAL-PET [66] and CALM-PD [67] trials compared clinical ratings of disease progression with measures of loss of putamen dopaminergic function in early PD cases randomized 1:1 to either levodopa or a dopamine agonist. The REAL-PET trial compared ropinirole- and levodopa-treated arms and used ^18^F-dopa PET as an imaging marker while the CALM-PD trial compared pramipexole- and levodopa-treated arms and used ^123^I-beta-CIT SPECT to follow disease progression. In both the trials, the imaging found that dopamine terminal function declined relatively more slowly in patients receiving an agonist compared with levodopa. However, clinically, the patients did better when taking levodopa. The ELLDOPA trial used ^123^I-beta-CIT SPECT to examine the relative rates of progression of *de novo* PD patients randomized to placebo, 150 mg, 300 mg, or 600 mg daily doses of levodopa [68]. Higher doses of levodopa were associated with faster declines in striatal ^123^I-beta-CIT uptake over 9 months but greater clinical benefit. These trials highlight the difficulties of employing imaging biomarkers to follow PD progression. It seems probable that exposure to oral levodopa over months has a direct depressant effect on striatal uptake of both ^18^F-dopa and beta-CIT thus erroneously giving the impression that disease is progressing faster [69].

Other studies of possible neuroprotective agents using imaging biomarkers have been negative and less controversial. The mixed JNK inhibitor CEP1347, the vitamin coenzyme Q10 designed to promote mitochondrial aerobic respiration, the neuroimmunophylline GPI1845, and the glutamate release inhibitor riluzole have all failed to influence PD progression based on both clinical ratings and imaging measures. Interestingly, CEP1347 had a mild and unsuspected depressant effect on striatal beta-CIT uptake [65].

Restorative approaches have also been monitored with functional imaging in PD trials. Several small open series and 2 larger double-blind control trials examined the efficacy of striatal implants of fetal midbrain cells. ^18^F-dopa PET consistently demonstrated that the grafts were functioning, increases in striatal dopamine storage capacity being seen in the actively treated cases within 6 months of surgery [70]. In one patient, an amphetamine challenge led to dopamine release from their unilateral putamen graft [71]. However, the response to bilateral fetal cell transplants was disappointing in 2 subsequent double-blind randomized control trials despite imaging evidence of graft function [72, 73]. Both trials failed to meet their primary endpoints, though in the Freed trial, a subanalysis showed younger cases had significantly improved by 12 months after engraftment. After a 3-year follow-up, all patients had clinically improved when rated with the UPDRS motor scale [74]. A problem, however, was the development of continuous graft–induced dyskinesias in a significant minority of these cases even after their oral medication was withdrawn.

An alternative restorative approach to cell transplantation is direct putamen infusions of growth factors such as glial-derived neurotrophic factor (GDNF). A small open pilot study initially reported that intraputamen infusions of GDNF were effective in reversing PD disability in advanced cases, and this was associated with a 25% increase in putamen ^18^F-dopa uptake [75]. In 2 larger double-blind controlled studies, however, the clinical responses to intraputamen infusions of GDNF were variable, only a minority of patients in the active arms deriving benefit despite the consistent finding that putamen ^18^F-dopa uptake increased in the actively treated patients [76, 77]. Another issue was dramatic placebo responses in a few of the cases. The GDNF and transplant trials both suggest that it is not enough simply to implant dopamine-producing cells or encourage DA terminal sprouting in the denervated PD striatum. The release of dopamine by implanted cells or new terminals may need to integrate into striatal circuitry or be close to postsynaptic receptors to be effective.

Neurturin is a neurotrophic protein with similar actions to GDNF which can be delivered to the striatum by infusing AAV2 viruses genetically engineered to express the neurturin gene. An initial 12-month double-blind sham-surgery–controlled trial assessing adeno-associated virus type 2 (AAV2)-neurturin injected into the putamen bilaterally failed to meet its primary endpoint but showed positive results in a subgroup of subjects followed for 18 months. In a second multicenter, double-blind trial over 15 months, the safety and efficacy of AAV2-neurturin delivered to the putamen and substantia nigra were tested in 58 patients with advanced PD [78]. There was no significant difference between the groups in the primary endpoint (change in motor subscore of the Unified Parkinson’s Disease Rating Scale in a practically defined off state) or in most secondary endpoints.

## Imaging Mechanisms of Motor Complications in PD

Within 3 years of beginning oral levodopa therapy, PD patients begin to experience motor complications. Progressive loss of striatal dopamine terminal function reduces the capacity of patients to convert exogenous levodopa into dopamine, store it in vesicles, and release it physiologically. As a consequence, patients develop symptoms of wearing off. Mean putamen ^18^F-dopa uptake has been reported to be 28% lower in PD patients with motor fluctuations compared to cases with a sustained treatment response [79]. Loss of striatal ability to take up and store dopamine leads to nonbiological swings in synaptic dopamine levels after oral levodopa with overstimulation and abnormal internalization of postsynaptic D1 and D2 receptors. This can result in both involuntary dyskinesias and unpredictable “off” periods. The benzamide radioligands ^11^C-raclopride and ^123^I IBZM bind reversibly to D2 receptors and compete with endogenous synaptic dopamine. Changes in their striatal binding provide a measure of swings in the synaptic level of dopamine. Using ^11^C-raclopride PET, it has been shown that patients who have a sustained response to oral doses of levodopa show a progressive slow increase in brain synaptic dopamine levels after taking this agent [80]. Patients with motor fluctuations show a rapid rise of brain dopamine that peaks which then falls back to baseline because of reduced striatal storage capacity. In PD patients who experience levodopa-induced peak dyskinesias, their severity has been reported to correlate with the increase in brain levels of dopamine induced by taking the levodopa prodrug [81].

While excessive dopaminergic stimulation is one mechanism of peak dyskinesia induction, postsynaptic mechanisms are also important. Adenosine A2A sites on the striatal neurons regulate the response of the “indirect” striatal–ventral thalamic pathway to dopamine stimulation. This pathway connects striatal neurons expressing D2 sites to the internal pallidum and ventral thalamus via the external pallidum and subthalamus. Using ^11^C-SCH442416 PET, a marker of A2A binding, it has been shown that levels of striatal A2A availability are normal in nondyskinetic PD patients while those with dyskinesias show an increased A2A availability [82]. Increased adenosine transmission dampens indirect pathway activity which acts to filter out unwanted movements so allowing involuntary movements to break through. The striatum also contains glutamate N-methyl-D-aspartate (NMDA) ion channels which are voltage gated. These ion channels contain a phencyclidine site which is only available to ligands when the ion channel is open and active. The PET tracer ^11^C-CNS5161 binds to the phencyclidine site, and it has been reported that when PD patients are actively experiencing mild dyskinesias, the striatal uptake of ^11^C-CNS5161 becomes abnormally increased [83]. This finding may help to explain why the NMDA ion channel blocker amantadine has antidyskinetic properties.

As dopamine terminals are lost in the striatum, significant amounts of levodopa may be taken up by the serotonin terminals still present where it is converted to dopamine. The serotonin terminals, however, are unable to store and release dopamine under physiological control, and this may also contribute to motor fluctuations. Using ^11^C-raclopride PET to measure synaptic dopamine changes after a levodopa challenge, it has been shown that administration of the HT1a agonist buspirone reduced striatal dopamine release and abolished drug-induced dyskinesias [84].

## Serotonergic Dysfunction and PD Tremor

Serotonergic function can be assessed by measuring levels of median raphe serotonin HT_1A_ autoreceptor binding in the midbrain, a marker of serotonergic cell body density, or by measuring the availability of serotonin transporters (SERT) on the nerve terminals present in the brainstem and on terminals in the basal ganglia and cortex. HT_1A_ sites are also expressed by pyramidal cell bodies in the limbic cortex. ^11^C-WAY100635 PET is an HT_1A_ marker and shows a 25% loss of median raphe binding in early PD [85]. Surprisingly, levels of raphe ^11^C-WAY100635 uptake do not correlate with the severity of depressive symptoms in PD but, interestingly, correlate inversely with the severity of rest tremor [86]. There was no correlation of raphe HT_1A_ availability with limb bradykinesia or rigidity. This suggests that pathology in the midbrain tegmentum may be relevant to the etiology of PD tremor as well as the nigrostriatal dopamine loss.

^11^C-DASB PET is a selective SERT marker. Loane and colleagues studied tremulous and akinetic-rigid groups of PD cases with ^11^C-DASB PET and found that the tremulous cases showed greater reductions in DASB binding in the raphe, basal ganglia, thalamus, and motor cortex [87]. Levels of raphe and basal ganglia ^11^C-DASB uptake correlated inversely with the severity of postural tremor but not rest tremor. ^123^I-FP-CIT SPECT is a marker not only of DAT binding in the striatum but also of SERT binding in the midbrain. Pasquini and colleagues reported that the severity of PD tremor in the PPMI series correlated with loss of raphe but not loss of striatal ^123^I-FP-CIT signal [88]. While these findings appear to implicate serotonergic loss in the etiology of PD tremor, an alternative explanation is that midbrain pathology affecting the median raphe is causing cerebellothalamic circuits to become dysfunctional resulting in tremor.

## The Role of Serotonergic, Noradrenergic, and Cholinergic Dysfunction in Nonmotor Problems of PD Patients

PET and SPECT studies designed to detect a role of serotonergic loss in PD depression have reported inconsistent findings. ^11^C-RTI 32 PET is a marker of noradrenaline (NART) and dopamine transporter (DAT) availability. Eight PD patients with a history of episodes of depression were compared with 12 cases with no such history but a similar level of locomotor disability [18]. Depressive features were rated with the Beck Depressive Index and anxiety with the State Trait Anxiety Inventory. The PD patients with a history of depression showed reduced thalamic and locus ceruleus ^11^C-RTI 32 binding, reflecting reduced NART availability, relative to PD cases with no history of depression. Lower ^11^C-RTI 32 uptake was also seen in their amygdala and ventral striatum, both limbic areas. These findings suggest that a tendency to depression in PD may be associated with reduced noradrenergic and dopaminergic function in limbic areas rather than serotonergic loss. Levels of anxiety also correlated with reduced ^11^C-RTI 32 binding in these areas. Some PD patients experience relief of depressive symptoms after taking levodopa, supporting the view that loss of limbic dopamine plays a role in their depression.

A subset of PD patients experience disabling tiredness similar to chronic fatigue syndrome. On examination, their locomotor disability is no worse than that of nonfatigued cases but they lack motivation. In one series, the fatigued cases showed significantly reduced ^11^C-DASB binding in the caudate, putamen, ventral striatum, thalamus, cingulate, and amygdala compared to nonfatigued cases [89]. No correlation was seen between levels of fatigue and levels of striatal or limbic ^18^F-dopa uptake. This suggests that chronic fatigue in PD is associated with reduced limbic serotoninergic function rather than with reduced dopaminergic function in the basal ganglia.

Sleep disorders in PD take the form of excessive daytime somnolence (EDS) and parasomnias including REM behavior disorder (RBD). EDS is particularly common in PD patients receiving dopamine agonists. It can manifest as sudden sleep attacks which are particularly dangerous if one is driving. Those cases with EDS have been reported to show a greater reduction in brainstem ^11^C-DASB uptake suggesting that the serotonergic arousal centers may be dysfunctional [16]. ^11^C-MeNER PET is a specific reboxitine marker of noradrenaline transporter availability. Around one-third of PD patients have symptoms of RBD in which they act out their dreams rather than becoming atonic. In PD, the locus ceruleus and thalamus show significant reductions in NART binding and these are most evident in those patients who have polysomnography-proven concomitant RBD [17].

The cholinergic system originates in the pedunculopontine nucleus (PPN), which sends projections to the thalamus, and in the nucleus basalis of Meynert (NBM), which sends projections to cortical and limbic areas. Brain acetylcholine esterase (AChE) activity can be measured with ^11^C-NMP4A, ^11^C-PMP, and ^11^C-donepezil PET. In PD, reductions in thalamic ^11^C-PMP levels can be detected which are most severe in cases with postural instability and gait disturbance (PIGD) [15]. Nondemented PD patients with mild cognitive impairment show reduced cholinergic function most evident in the parietal and occipital cortex [90]. As cognitive function declines, the cholinergic deficit spreads forward to also involve the frontal cortex and becomes global in PD dementia. Levels of cortical ^11^C-PMP uptake in PD correlate with their MMSE ratings and performance on executive tests such as card sorting and trail making [15]. They do not, however, correlate with severity of locomotor disability rated with the UPDRS. Presynaptic cholinergic terminal function can be assessed with both ^123^I-benzovesamicol (I-BV) SPECT and ^18^F-fluoroethoxy-benzovesimacol (FEOBV) PET, markers of vesicular transporter availability. Nondemented PD patients show focally reduced ^123^I-BV uptake in the parietal and occipital cortex which again spreads to become global as cognitive function declines [91].

## Imaging Mechanisms of Cognitive Deficit in PD

### Magnetic Resonance Imaging

80% of PD patients will develop dementia if they live for 20 years or longer [19] because of a combination of cortical Lewy body disease, degeneration of dopaminergic and cholinergic projections to cortical areas, small-vessel disease, and concomitant Alzheimer pathology. Volumetric MRI shows cortical thinning, even in early PD cases with no overt cognitive dysfunction [92], and cortical thickness correlates with MOCA scores [93]. BOLD resting fMRI detects oscillations in brain venular oxygenation, and these are synchronized in regions that are functionally connected. In PD, resting fMRI has shown reduced connectivity in attentional and executive networks and the default mode network—this subject area has been recently reviewed [4, 94, 95].

A meta-analysis performed by Wolters and colleagues [95] on studies using either seed-based or independent component (ICA) analyses concluded that cognitively impaired Parkinson’s cases (PD-CI) showed reduced connectivity of their right Rolandic operculum, left inferior parietal gyri, right angular gyrus, right calcarine fissure, left parahippocampal gyrus, right superior frontal gyrus, and right precentral gyrus compared to healthy controls (HC) while functional connectivity was increased in the right supramarginal gyrus. When cognitively unimpaired PD cases (PD-CU) were compared with PD-CI, the latter showed relatively lower connectivity in the left precuneus, right median cingulate gyrus, left superior frontal gyrus, and right precentral gyrus and increased functional connectivity in the right cerebellum (hemispheric lobule VI). These workers concluded that, across the 17 resting fMRI series included, the most consistent finding in PD-CI was reduced connectivity of the default mode network (DMN) and suggested this may provide a predictor of subsequent dementia.

Baggio and coworkers [4] have reported reduced resting DMN connectivity in their PD-CU cases receiving treatment. Seed-to-seed analyses showed that the worse cognitive status was associated with reduced connectivity within the DMN and the dorsal attentional network (DAN). There was also reduced coupling between the DAN and the frontoparietal network (FPN). Szewczyk-Krolikowski and colleagues have used ICA to compare resting functional connectivity in PD patients taken off medication compared with those taking medication and healthy controls [96]. The “off” cases showed reduced connectivity between the basal ganglia and widespread cortical regions and the brainstem. The treated cases had normal brain connectivity.

Although resting state functional MRI opens the door to studying the functional effects of drug treatment, deep-brain electrical and focused ultrasound stimulation, and noninvasive transcranial magnetic and alternating current stimulation, on brain connectivity, it has its difficulties. To date, results of fMRI studies on PD have been inconsistent possibly because of small study samples, different methods of image preprocessing and analysis, and artefacts due to head motion.

### ^18^F-FDG PET

In nondemented PD patients, covariance analysis reveals an abnormal profile of relatively increased lentiform nucleus and reduced frontoparietal glucose metabolism that has been labeled the “PD-related profile” (PDRP) [55]. Its degree of expression correlates with levels of motor disability rated with the UPDRS [56]. As cognitive deficits develop in PD, covariance analysis can also identify an independent cognitive metabolic PDCP profile characterized by hypometabolism of dorsolateral prefrontal and premotor cortex, rostral supplementary motor area (preSMA), precuneus, and posterior parietal regions, associated with relative metabolic increases in the cerebellum [55, 97]. Expression of the PDCP in individual PD cases correlates with their performance on the California Verbal Learning Test.

PD dementia (PDD) and dementia with Lewy body (DLB) have similar underlying cortical and brainstem Lewy pathology but are differentiated by the timing of onset of the dementia. This onset is at least 1 year after the onset of parkinsonism in PDD but before or less than 1 year after the onset of parkinsonism in DLB. PDD and DLB both show cortical atrophy on MRI, but whereas Alzheimer’s disease targets the mesial temporal lobe and hippocampus, DLB targets the insula and posterior parietal cortex and spares the mesial temporal cortex and hippocampus [98]. In pathologically confirmed cases, it was reported that the presence of mesial temporal atrophy distinguished AD from DLB with 91% sensitivity and 94% specificity [99]. However, these were well-established cases.

PDD and DLB patients show a similar pattern of reduced brain rCMRGlc which targets the parietal and temporal association areas similar to Alzheimer’s disease, but there is a significantly greater level of occipital hypometabolism in pathologically proven DLB cases [97, 100]. Occipital hypometabolism has been reported to distinguish DLB from AD with a sensitivity of 83 to 99% and specificity between 71 and 93% [101, 102]. Additionally, while the precuneus is hypometabolic, there is an island of preserved posterior cingulate metabolism in DLB [103]. PDD cases show a global cortical cholinergic deficit with the acetylcholine esterase markers ^11^C-MP4A and ^11^C-PMP PET and with the cholinergic vesicle transporter marker ^123^I-BV (benzovesamicol) SPECT [15, 90, 91]. Levels of cortical ^11^C-PMP PET uptake in PDD correlate with MMSE scores but not with locomotor disability rated with the UPDRS [104]. DLB subjects have been scanned with both ^123^I-BV SPECT and FEOBV PET and also showed global cortical and thalamic loss of cholinergic vesicle transporter binding [105].

**Fig. 3:**
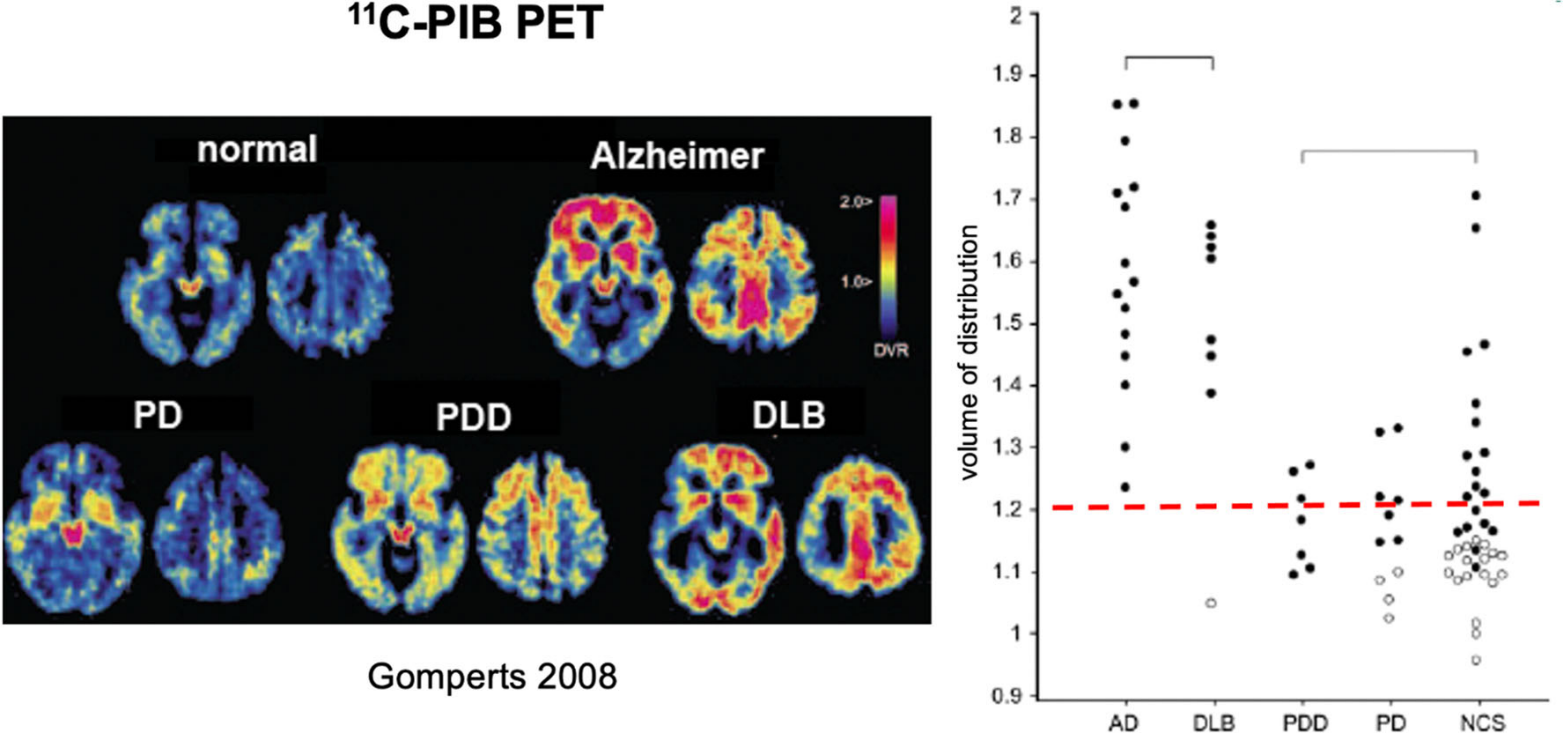
Imaging β-amyloid load in Lewy body and Alzheimer diseases (picture from ref [108]: Gomperts et al. 2008)

Pathological studies have revealed that both end-stage DLB and PDD cases have a significant prevalence of coincident Alzheimer pathology [106]. PIB is a neutral benzothiazole analogue of the histological dye thioflavin-T and binds to extracellular beta sheeted β-amyloid fibrillar plaques present in Alzheimer’s disease. ^11^C-PIB PET has been used to estimate the prevalence of raised β-amyloid deposition in nondemented PD patients (PDND), PD cases with later-onset dementia (PDD), and cases with DLB. To date, PDND cases have shown a similar prevalence of incidental amyloid to healthy age-matched controls [107, 108]. Around half of PDD cases scanned have shown increased ^11^C-PIB uptake suggesting that amyloid pathology is not a major contributor to the cognitive problems in many cases. In contrast, 70 to 80% of dementia with Lewy body (DLB) patients, in which onset of dementia starts before or coincides with parkinsonism, show increased cortical ^11^C-PIB signal approaching Alzheimer levels (Fig. 3).

A consecutive series of 18 probable DLB cases at the Mayo Clinic has been studied with both tau tangle imaging (^18^F-flortaucipir PET) and ^11^C-PiB PET [109]. Findings were compared with 18 Alzheimer cases matched for age and sex and a large healthy control database. The AD cohort had greater ^18^F-flortaucipir uptake than DLB cases, and medial temporal lobe tau was only seen in AD. Twelve of the 18 DLB cases showed raised posterior parietal, temporal, and occipital tau signal, and 8 of these cases had β-amyloid deposition. This suggests that both Lewy body and Alzheimer pathologies can underlie the entity clinically labeled as DLB explaining its aggressive nature. Unlike Alzheimer’s disease, in which the cortical tau is only seen when cortical β-amyloid is also present, DLB cases can have cortical tau tangles in the absence of amyloid aggregates. Gomperts and colleagues collected 24 patients with Lewy body disorders (7 DLB, 8 PD-MCI, and 9 PD-cognitively normal) and compared their ^18^F-flortaucipir PET, ^11^C-PiB PET, and MRI findings with those of a group of 29 controls with minimal brain amyloid present on ^11^C-PiB PET [110]. A minority of the PD-MCI and DLB cases individually showed a raised tau load, and this targeted the inferior temporal lobe and the precuneus. 17 Lewy body cases had raised amyloid levels, but again, raised tau signal did not appear to correlate with the presence of amyloid plaques. While MMSE and CDR SOB ratings correlated with inferior temporal and precuneus tau load, they were not influenced by β-amyloid load. The authors concluded that tau deposits are common in DLB and PD-MCI, can arise in the absence of significant amyloid burden, and contribute to cognitive impairment.

## Discriminating DLB from Alzheimer’s Disease

These 2 dementias can be difficult to discriminate clinically as most DLB cases also have Alzheimer pathology at postmortem [111]. DLB is characterized by the presence of fluctuating cognition, the presence of parkinsonism, visual hallucinations, and REM sleep behavior disorder. However, these features are not always present and can occasionally be seen in Alzheimer’s patients. Additionally, as these dementias also have associated small-vessel pathology, this vascular disease can complicate the clinical picture. It has been estimated that around 20% of clinical diagnoses of DLB using the consensus criteria alone are incorrect. The presence of hippocampal atrophy on MRI discriminates 90% of Alzheimer from DLB cases in established disease, but hippocampal atrophy is not specific for AD and can be difficult to assess visually, and the more clinically severe DLB cases can have significant levels of AD pathology present. Occipital lobe hypometabolism on FDG PET discriminated over 80% of DLB from AD cases in a pathologically validated series [100, 112]. The most sensitive discriminator, however, validated against postmortem pathology, is DAT imaging with ^123^I-FP-CIT SPECT (DaTSCAN) or ^18^F-FP-CIT PET. In one series, ^123^I-FP-CIT SPECT discriminated pathologically proven DLB from AD with 88% sensitivity and 100% specificity as the latter is not associated with loss of striatal DAT binding [113, 114]. However, occasionally, AD with small-vessel disease causing a loss of striatal DAT binding can mimic DLB. In this situation, MIBG scintigraphy, a marker of myocardial sympathetic innervation, can be helpful. PD and DLB cases both show reduced MIBG heart to mediastinal (*H*:*M*) ratios whereas these are normal in AD even if small-vessel disease is present in the brain [115]. MIBG scintigraphy has been reported to show 94% specificity for DLB compared with AD.

## Atypical Parkinsonian Syndromes

### Multiple System Atrophy

Multiple system atrophy (MSA) is an atypical sporadic form of neurodegenerative parkinsonism which is associated with autonomic failure and ataxia. Depending on whether parkinsonism or cerebellar ataxia is predominant, the motor phenotypes are labeled MSA-P and MSA-C. The pathology is characterized by argyrophilic glial inclusions containing aggregated α-synuclein and ubiquitin which target the substantia nigra compacta and the putamen along with the pons, the cerebellar nuclei, and the intermediolateral columns of the spinal cord.

**Fig. 4:**
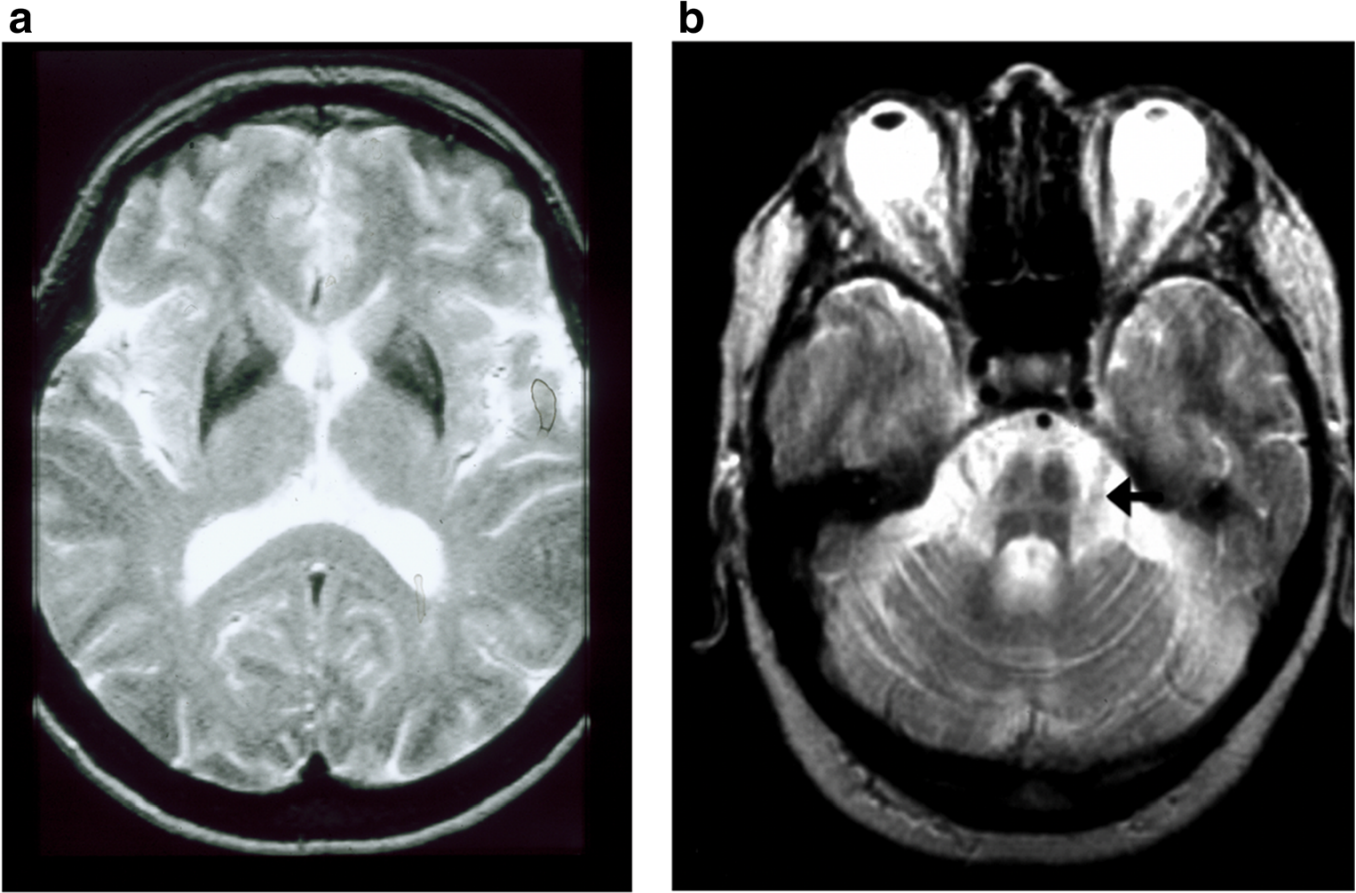
(a) T2-weighted MRI showing a low lateral putamen signal at the same level as the pallidum in MSA. (b) T2-weighted MRI showing the pontine hot cross bun sign in MSA

MSA pathology leads to reduced putamen signal on T2-weighted MRI because of iron deposition. This can be accompanied by a lateral rim of increased signal because of associated gliosis [116] (Fig. 4a). Degeneration of the pons causes the lateral as well as longitudinal pontine fibers to become evident as high signal manifesting as the “hot cross bun” sign [116, 117] (Fig. 4b). These features, while specific for MSA, are not sensitive and are usually only visually obvious on MRI in advanced cases. Diffusion tensor imaging (DTI) increases the sensitivity of MRI for detecting specific MSA changes. Increased putamen water diffusivity and reduced directionality “anisotropy” of water flow are seen in both the putamen and middle cerebellar peduncles in a majority of cases [118, 119]. Water diffusivity is normal in the putamen and middle cerebellar peduncles of PD cases.

**Fig. 5:**
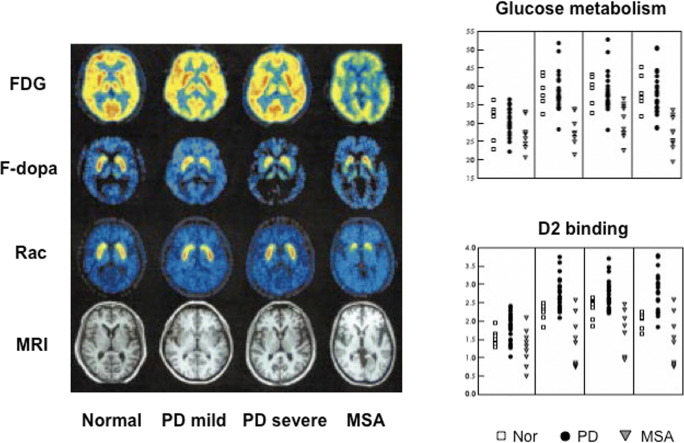
Glucose metabolism, dopamine storage capacity, and D2 receptor binding in parkinsonian syndromes. (Picture from ref [122]: Eckert et al. 2005)

^18^FDG PET studies in MSA show reduced levels of striatal, pontine, and cerebellar glucose metabolism in contrast to PD in which these are all preserved [120, 121] (Fig. 5). The presynaptic dopaminergic system is affected by MSA pathology in a similar way to Lewy body pathology so that, while ^18^F-dopa PET, VMAT2, and DAT imaging can separate PD and MSA from healthy controls, they cannot reliably discriminate these 2 parkinsonian conditions [123]. Unlike PD, MSA is associated with a loss of putamen dopamine D2 binding but this is relatively mild and does not provide a sensitive discriminator of MSA from PD [124]. In one series, ^123^I-IBZM SPECT reported reduced striatal D2 binding in two-thirds of *de novo* parkinsonian patients who had a negative apomorphine response and were thought to have possible early MSA [125].

**Fig. 6:**
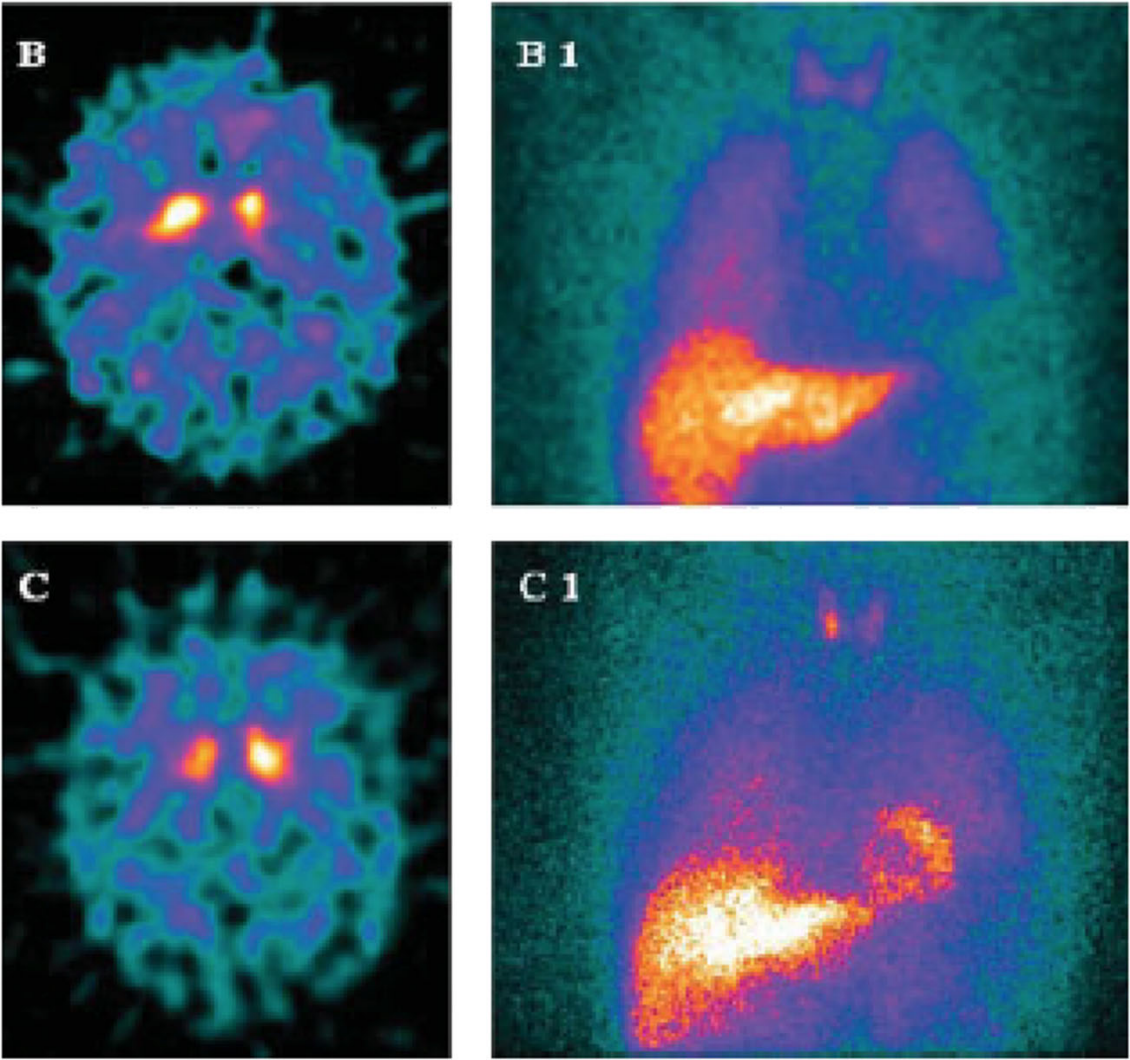
FP-CIT SPECT and ^123^I-MIBG scintigraphy images of cardiac sympathetic function in PD (upper) and MSA (lower). In MSA, the myocardial MIBG signal is intact. (Picture from Novellino et al. Movement Disorders 2009; 24(15):2242–2248)

^123^I-MIBG SPECT is a marker of adrenergic terminal function and can be used to study functional integrity of cardiac sympathetic innervation in MSA and PD (Fig. 6). Mediastinal ^123^I-MIBG signal is reduced in a majority of PD cases [126] whereas ^123^I-MIBG SPECT is normal in a majority of MSA cases [127] as the autonomic dysfunction results from loss of pre- rather than postsynaptic innervation. Use of ^123^I-MIBG SPECT can, thus, help differentiate MSA from PD.

α-Synuclein has been imaged in multiple system atrophy GCIs with ^11^C-BF227 (2-[2-(2-dimethylaminothiazol-5-yl)ethenyl]-6-[2-(fluoro)ethoxy] benzoxazole) PET [128]. BF227 stains Asyn-containing GCIs in postmortem brains. Eight MSA cases were compared with age-matched normal controls. PET detected raised signal in the subcortical white matter (*p* < 0.001), putamen and posterior cingulate cortex (*p* < 0.005), globus pallidus, primary motor cortex and anterior cingulate cortex (*p* < 0.01), and substantia nigra (*p* < 0.05) in MSA cases compared to the normal controls. The MSA signals, however, were only raised by around 10%. The authors concluded that ^11^C-B227 PET was a promising surrogate marker for intracellular α-synuclein deposition in living brains. However, they failed to mention that it has a strong affinity for β-amyloid plaques as well as intracellular Asyn fibrils.

### Progressive Supranuclear Palsy

Progressive supranuclear palsy (PSP) is an atypical sporadic parkinsonian syndrome characterized clinically by early axial rigidity, postural instability and falls, and impairment of voluntary eye movements. Frontal dementia, nonfluent aphasia, and apraxia of limbs and speech can also be features. There are now 8 recognized phenotypes of PSP: Richardson’s syndrome (PSP-RS) is characterized by l-dopa-resistant parkinsonism and oculomotor apraxia. Other phenotypes are PSP with predominant parkinsonism (PSP-P), pure akinesia and gait freezing (PSP-PAGF), corticobasal syndrome (PSP-CBS), predominant language dysfunction and speech apraxia (PSP-PNFA and PSP-AOS), predominant frontotemporal dysfunction (PSP-FTD), parkinsonism and cerebellar ataxia (PSP-C), and parkinsonism with primary lateral sclerosis (PSP-PLS). The characteristic pathology of PSP is intraneuronal globose linear neurofibrillary tangles containing 4-repeat tau found in the substantia nigra, basal ganglia, oculomotor nuclei, superior colliculi, brainstem nuclei, periaqueductal gray matter, and premotor cortex. Tau-immunoreactive tufted astrocytes are also found in these regions.

Most imaging studies have been carried out on the Richardson phenotype of PSP. In contrast to MSA which targets the striatum, PSP patients do not show reduced putamen T2-weighted MRI signal changes. Established cases have third ventricular widening and midbrain atrophy evident on sagittal views—sometimes known as the “hummingbird” sign [116]. These MRI changes are generally only evident with established cases. The sensitivity of MRI for discriminating PSP from PD can be improved by the use of DTI which detects reduced fractional anisotropy in the putamen of both PSP and MSA cases [118]. In contrast to MSA, the superior rather than the middle cerebellar peduncle shows increased diffusivity in PSP enabling these 2 conditions to be discriminated [129].

In PSP, FDG PET reveals hypometabolism of the premotor cortex, thalamus, basal ganglia, midbrain, and cerebellar dentate nuclei. Levels of FDG uptake correlate inversely with disease duration and performance on psychometric tests of frontal function [123]. The presence of reduced striatal metabolism sensitively discriminates 90% of PSP cases from PD; however, as striatal hypometabolism is also a feature of MSA, striatal FDG uptake alone cannot reliably discriminate between these 2 atypical parkinsonian conditions [122]. As with MSA, striatal D2 binding is reduced in more advanced PSP cases and this can be detected with ^11^C-raclopride PET [124] or IBZM SPECT [130].

Tau imaging agents now exist which sensitively detect the intraneuronal paired helical filament tangles found in Alzheimer’s disease. These include ^18^F-flortaucipir, ^18^F-MK-6240, ^18^F-PI-2620, and ^11^C-PPB3 PET. Controversy exists over whether these tracers can also detect 4R-linear tau tangles. The first 3 PET tracers, ^18^F-flortaucipir, ^18^F-MK-6240, and ^18^F-PI-2620, all show increased uptake in the pallidum and cortex of probable PSP cases. However, postmortem autoradiography has found little evidence that flortaucipir binds to linear tau [131]. It was thought that flortaucipir might be binding to MAOB, but blockade with deprenyl had no effect, so this appears not to be the case. It is possible, however, that these tracers are binding to other proteins associated with the linear tau tangles. In contrast, ^11^C-PPB3 appears to bind to linear as well as paired helical tau tangles and so ^11^C-PPB3 PET provides a potential linear tau marker [132]. ^11^C-PPB3, however, is difficult to make and use as it is subject to radiolysis and isomerization in visible light.

### Corticobasal Degeneration

The corticobasal syndrome presents with an akinetic-rigid, apraxic limb which may exhibit alien behavior. Cortical sensory loss, dysphasia, myoclonus, supranuclear gaze problems, and bulbar dysfunction are all features while intellect is relatively spared until late. Eventually, all 4 limbs become asymmetrically involved and the condition is poorly l-dopa responsive.

Corticobasal degeneration, like PSP, is a 4R repeat tauopathy associated with linear tau tangles. The pathology consists of collections of swollen, achromatic, tau +ve staining Pick cells in the absence of argyrophilic Pick bodies which target the posterior frontal, inferior parietal, and superior temporal lobes, the substantia nigra and the cerebellar dentate nuclei. Tau inclusions are also seen in glia with astrocytic plaques and thread-like pathology in white and grey matter. Tau-positive globose neurofibrillary tangles are common in the substantia nigra and locus ceruleus while ballooned achromatic neurons are common in affected cortical regions.

A number of clinical pathologies can produce corticobasal syndrome mimicking corticobasal degeneration including PSP, Lewy body disease, multi-infarct disease and multifocal leukoencephalopathy, frontotemporal dementia, and prion and Alzheimer’s disease. In corticobasal degeneration (CBD), asymmetric hemispheric atrophy may be present on structural imaging and MRI can detect vascular and demyelinating diseases.

PET and SPECT studies on patients with the corticobasal syndrome (CBS) have shown significant asymmetric reductions in resting cortical oxygen and glucose metabolism in posterior frontal, inferior parietal, and superior temporal regions [133, 134]. The thalamus and striatum are also asymmetrically involved, metabolic reductions being most severe contralateral to the more affected limbs. This contrasts with PD patients who have preserved levels of striatal and thalamic glucose metabolism.

Striatal ^18^F-dopa uptake is reduced in CBS in an asymmetrical fashion, being most depressed contralateral to the more affected limbs [133]. Like PSP, but in contrast to PD, head of caudate and putamen ^18^F-dopa uptake are similarly depressed in CBS. ^123^I-beta-CIT SPECT also shows an asymmetrical reduction in striatal dopamine transporter binding in CBS while ^123^I-IBZM SPECT shows a severe asymmetrical reduction of striatal D2 binding [135].

The above imaging findings may help discriminate CBD from Pick’s disease in which inferior frontal hypometabolism predominates, from PD in which striatal metabolism is preserved and head of caudate ^18^F-dopa uptake is relatively spared, and from PSP in which frontal and striatal metabolism tend to be more symmetrically involved. Having said that, both Pick’s and PSP pathology can be associated with clinical CBS.

Tau PET (^18^F-flortaucipir) has been performed on amyloid-negative CBS cases, one of which was an autopsy-confirmed case of CBD [136–139]. Increased signal was seen in regions targeted by CBD pathology—the substantia nigra, brainstem, globus pallidus, and posterior frontal, inferior parietal, and temporal cortical areas. In the postmortem case, the raised ^18^F-flortaucipir signal corresponded to regions of increased linear tau tangles. However, direct binding of ^18^F-flortaucipir to linear 4R tau was not autoradiographically studied and the signals were lower than those seen in Alzheimer’s disease. Currently, tau PET cannot be used to discriminate the 4R-repeat tauopathies PSP and CBD. Whether tau PET is binding to linear tau or an associated protein is still under debate.

## Utility of FDG PET for Discriminating Between Atypical Parkinsonian Syndromes

A number of reports have presented approaches for optimizing the use of FDG PET for discriminating PD, DLB, MSA, PSP, and CBD cases by generating individual statistical parametric maps (SPM) for visual inspection or automated statistical interrogation. Caminiti and colleagues generated individual t maps of abnormal FDG uptake relative to age-matched normals for each possible atypical Parkinsonian syndrome (APS) case at baseline, and a visual diagnosis was assigned by viewers unaware of the clinical presentation [140]. The FDG PET diagnosis was then compared with long-term clinical follow-up as the standard of truth (SOT). These workers found that SPM t-map classification showed 98% sensitivity, 99% specificity, and 99% accuracy for discriminating APS from PD and that there was a significant agreement with the clinical diagnosis at follow-up (*p* < 0.001).

Eidelberg and colleagues have used FDG PET brain imaging combined with an automated image classification algorithm to classify parkinsonian patients as PD or APS at the time when the clinical diagnosis was still uncertain [141]. Two cohorts were considered—one clinically diagnosed by movement disorder specialists following consensus criteria 2 years after imaging—which was the standard of truth—and one diagnosed by general neurologists not necessarily following consensus criteria 2 years after imaging. Against the SOT, image-based automated classification of the first cohort resulted in 86.0% sensitivity and 92.3% specificity for discriminating PD and 84.6% sensitivity and 97.7% specificity for detecting APS. Compared with the general neurologists’ diagnoses, imaging achieved 94.7% sensitivity and 83.3% specificity for PD and 88.2% sensitivity and 76.9% specificity for APS. These workers concluded that the automated imaging algorithm could improve accurate diagnosis of PD by 10 to 15% and of APS by 20% in the absence of a movement disorder expertise.

Meyer and coworkers have performed a meta-analysis of the role of FDG PET for the differential diagnosis of Parkinsonism [97]. They estimated that the diagnostic sensitivity and specificity for visual PET readings supported by voxel-based statistical analyses for diagnosis of atypical parkinsonian syndromes combined were 91.4% and 90.6%, respectively. The diagnostic specificity of FDG PET for diagnosing multiple-system atrophy, progressive supranuclear palsy, and corticobasal degeneration was consistently high (> 90%), but the sensitivity was more variable (> 75%) on the borderline of clinically valuable.

## Conclusions

In Parkinson’s disease, structural changes can now be detected in the substantia nigra compacta with MRI using melanin-sensitive sequences and with diffusion tensor imaging (DTI). Disease progression can be detected as neuromelanin is lost and the nigral free water pool increases with time. Resting functional BOLD MRI can be used to examine changes in brain connectivity over time in PD and detects impaired function of executive and attentional networks. The atypical parkinsonian syndromes MSA, PSP, and CBD can be discriminated from idiopathic PD using DTI by the presence of abnormal putamen water diffusivity. Additionally, DTI can discriminate MSA from PSP as the middle cerebellar peduncles show increased diffusivity in MSA while the superior cerebellar peduncles are targeted in PSP.

Radiotracer-based PET and SPECT imaging provide a sensitive and objective means of detecting dopamine terminal dysfunction in PD and DLB, and the use of DAT SPECT or PET can support a diagnosis of these conditions in which doubt exists and rationalize the use of dopamine replacement therapy. The loss of striatal dopaminergic function in late-onset sporadic disease targets the putamen in an asymmetrical fashion and spares the head of caudate. In dominantly inherited genetic forms of PD, such as those associated with GBA or LRRK2 mutations, a similar pattern of loss of striatal dopaminergic function is seen. Recessively inherited PD cases, however, show more severe and widespread striatal dopamine terminal dysfunction. MIBG SPECT can detect reduced cardiac adrenergic innervation in PD and DLB cases, discriminating them from essential tremor and Alzheimer’s disease.

FDG PET measures brain glucose metabolism and shows a loss of putamen signal in atypical PD variants, discriminating them from idiopathic PD in which putamen metabolism is spared. The use of covariance analysis with FDG PET reveals a PD-related profile (PDRP) with relatively raised striatal and lowered frontal and parietal metabolism. Expression of this PDRP correlates with disease severity and can be used to follow disease progression or treatment efficacy. While MIBG SPECT detects reduced cardiac sympathetic innervation in PD cases, this is not a feature of MSA, PSP, or CBD cases.

When present, subclinical dopamine terminal dysfunction can be detected with PET and SPECT in subjects at high risk for PD. These subjects include GBA, LRRK2, and parkin mutation carriers, subjects with a family history of PD, cases of late-onset idiopathic hyposmia, and patients with idiopathic REM sleep behavior disorder (RBD). Identification of subclinical Parkinson’s disease could potentially help identify cases for trials of neuroprotective therapies if an effective and safe intervention is identified.

Imaging potentially provides an objective biomarker for monitoring PD progression and the efficacy of putative neuroprotective and restorative approaches. However, trials have been confounded by the direct effects of interventions on the imaging modalities making interpretation of their findings difficult at times.

Finally, nonmotor complications of PD, such as dementia, depression, and sleep disorders, are a major problem that can influence quality of life to a greater degree than locomotor disability. Imaging can help understand the mechanisms underlying these complications and so help rationalize therapeutic approaches. Dementia is multifactorial involving cortical Lewy body pathology, loss of frontal dopaminergic and cortical cholinergic projections, and concomitant Alzheimer pathology. Sleep disorders are associated with loss of serotonergic (EDS) and noradrenergic (RBD) projections. There may come a time when, with the help of imaging, personalized medicine becomes a reality in parkinsonian syndromes.

## Supplementary Information

ESM 1 (PDF 1224 kb)
